# Cellular senescence and metabolic aging in type 2 diabetes: mechanistic insights and translational implications

**DOI:** 10.3389/fendo.2026.1799261

**Published:** 2026-03-23

**Authors:** Ammaar Riyaz Syed, Radwan Abdulaziz Aloti, Bassam Jehad Awad, Wasim Iyad Ibrahim Alghoul, Mohamed Ayman Mohamed Samy Aly Hassan, Ahmad Kharoufeh, Imran Rangraze

**Affiliations:** Department of Internal Medicine, Ras Al Khaimah (RAK) Medical and Health Science University, Ras Al Khaimah, United Arab Emirates

**Keywords:** glycemic control, inflammaging, insulin resistance, SASP, SCAP, senescence, senolytics, type 2 diabetes

## Abstract

**Background:**

Type 2 diabetes mellitus (T2DM) is traditionally conceptualized as a disorder of insulin resistance and β-cell dysfunction driven by metabolic overload. Increasing evidence now implicates cellular senescence—a stress-induced state of durable cell-cycle arrest accompanied by a pro-inflammatory senescence-associated secretory phenotype (SASP)—as a biologically distinct contributor to metabolic dysfunction. Senolytic therapies, which selectively eliminate senescent cells by targeting senescent cell anti-apoptotic pathways (SCAPs), have therefore emerged as potential disease-modifying interventions.

**Methods:**

We conducted a structured narrative review of preclinical and human studies published between 2010 and 2025, identified through PubMed, Scopus, and Embase. Evidence was synthesized qualitatively, with emphasis on mechanistic plausibility, tissue specificity, translational readiness, and safety considerations relevant to T2DM and its complications.

**Results:**

Preclinical studies consistently demonstrate that clearance of senescent cells in adipose tissue, liver, and pancreatic islets improves insulin sensitivity, attenuates SASP-mediated inflammation, and preserves β-cell function across multiple diabetic models. Human evidence remains limited to small, short-duration pilot studies primarily designed to assess biological target engagement and short-term safety. These studies report reductions in senescence markers and inflammatory mediators following intermittent senolytic exposure, but lack standardized metabolic endpoints and long-term follow-up.

**Conclusion:**

Senolytic therapy represents a biologically compelling yet still experimental strategy for addressing the intersection of metabolic dysfunction and biological aging in T2DM. By reframing T2DM as a disease of metabolic aging, this review positions cellular senescence as an upstream therapeutic target and provides a conceptual framework to guide future precision-based clinical trials, rather than advocating near-term clinical adoption. By integrating geroscience with diabetology, this review positions cellular senescence as a biologically upstream framework that may inform future precision-based and disease-modifying strategies in type 2 diabetes.

## 1 Introduction

Type 2 diabetes mellitus (T2DM) is a progressive metabolic disorder characterized by insulin resistance, β-cell dysfunction, and chronic low-grade inflammation. Despite major therapeutic advances, current treatment strategies primarily address downstream metabolic abnormalities and rarely alter the underlying trajectory of disease progression (1–3). This limitation has prompted increasing interest in upstream biological mechanisms that link metabolic stress to irreversible tissue dysfunction (4).

Cellular senescence has emerged as a unifying biological process connecting aging, obesity, and metabolic disease. Senescent cells accumulate in adipose tissue, liver, skeletal muscle, and pancreatic islets—organs central to glucose homeostasis—where persistent secretion of pro-inflammatory and matrix-remodeling factors collectively termed the senescence-associated secretory phenotype (SASP) disrupts insulin signaling, promotes immune cell infiltration, and accelerates β-cell failure (5). Importantly, even a modest burden of senescent cells can exert disproportionate systemic effects, positioning senescence as a mechanistically distinct contributor rather than a secondary consequence of metabolic dysfunction (6).

While several prior reviews have described associations between senescence and metabolic disease, they have often treated senescence as an epiphenomenon of obesity or aging, or have focused narrowly on individual tissues or molecular pathways. In contrast, the present review advances the field by explicitly reframing T2DM as a disorder arising from the convergence of metabolic stress and biological aging (6). This perspective distinguishes mechanistic plausibility from clinical efficacy, integrates senolytic and senomorphic strategies within a unified disease framework, and critically evaluates human evidence within appropriate translational boundaries.

Rather than proposing senolytics as near−term therapeutic agents, this review establishes a conceptual framework in which type 2 diabetes mellitus is understood as a convergence of metabolic stress and biological aging. This perspective positions cellular senescence as an upstream disease−organizing process that informs future precision−based translational research.

While this review references non-diabetic aging models where mechanistically informative, interpretation remains centered on metabolic disease–specific data relevant to T2DM pathophysiology.

## 2 Methodology

This article is a structured narrative review designed to synthesize mechanistic, preclinical, and early translational human evidence linking cellular senescence to type 2 diabetes mellitus (T2DM).

### 2.1 Search strategy and study identification

A comprehensive literature search was conducted using PubMed, Scopus, and Embase covering publications from January 2010 to September 2025. Search terms included combinations of: “cellular senescence,” “senolytics,” “senescence-associated secretory phenotype,” “SASP,” “SCAP,” “type 2 diabetes mellitus,” “insulin resistance,” and “β-cell dysfunction.”

The initial search yielded 1,274 records (PubMed: 512; Scopus: 438; Embase: 324). After removal of duplicates (n=243), 1,031 records underwent title and abstract screening. Of these, 312 articles were selected for full-text review based on relevance to metabolic disease and senescence biology. Ultimately, 148 primary studies were included in the qualitative synthesis, comprising mechanistic *in vitro* studies, animal models, early-phase human studies, and organ-specific clinical investigations.

### 2.2 Inclusion and exclusion criteria

Included studies met the following criteria:

Original *in vitro*, *in vivo*, or human research

Direct evaluation of senescence mechanisms or senolytic interventions relevant to metabolic dysfunction

Published in English between 2010–2025

Excluded:

Narrative reviews, editorials, conference abstracts

Studies not addressing metabolic endpoints or senescence biology

Non-English publications

Dual independent screening was not formally performed given the narrative review design; however, all included studies were cross-validated by senior authors to ensure thematic consistency and scientific rigor.

### 2.3 Evidence synthesis and methodological limitations

Given the heterogeneity of experimental models, endpoints, and clinical designs, quantitative meta-analysis was not feasible. Evidence was therefore synthesized qualitatively with emphasis on mechanistic plausibility, tissue specificity, translational readiness, and safety considerations.

We acknowledge that, as a structured narrative review, this approach does not incorporate formal risk-of-bias assessment or PRISMA-based systematic methodology. Accordingly, conclusions are interpretative and hypothesis-generating rather than definitive.

Study selection and thematic synthesis were independently reviewed by multiple authors. Any discrepancies regarding inclusion eligibility or interpretive emphasis were resolved through structured discussion and consensus among senior investigators. While formal inter-rater reliability metrics were not calculated due to the narrative design, cross-validation was undertaken to ensure conceptual consistency and balanced interpretation of evidence.

## 3 Biology of cellular senescence and metabolic disease

### 3.1 Cellular senescence as a biological stress response

Cellular senescence is a stress-induced state of durable cell-cycle arrest triggered by telomere attrition, DNA damage, oxidative stress, mitochondrial dysfunction, or oncogenic signaling (7). While initially protective—limiting malignant transformation and facilitating tissue remodeling—persistent senescence contributes to age-related tissue dysfunction when clearance mechanisms fail (8, 9). Senescent cells are characterized by activation of cell-cycle inhibitors (p16^INK4a, p21^Cip1), altered metabolism, and secretion of SASP factors that remodel local and systemic environments (10).

### 3.2 Link between senescence, metabolic dysfunction, and the rationale for senolytics

Cellular senescence represents a stress-induced state of irreversible cell-cycle arrest accompanied by sustained metabolic activity and inflammatory signaling (5). In metabolically active tissues, the pathological relevance of senescence lies not merely in growth arrest, but in the acquisition of a robust senescence-associated secretory phenotype (SASP), which exerts paracrine and systemic effects on insulin signaling and tissue homeostasis (6, 11).

**Figure 1:**
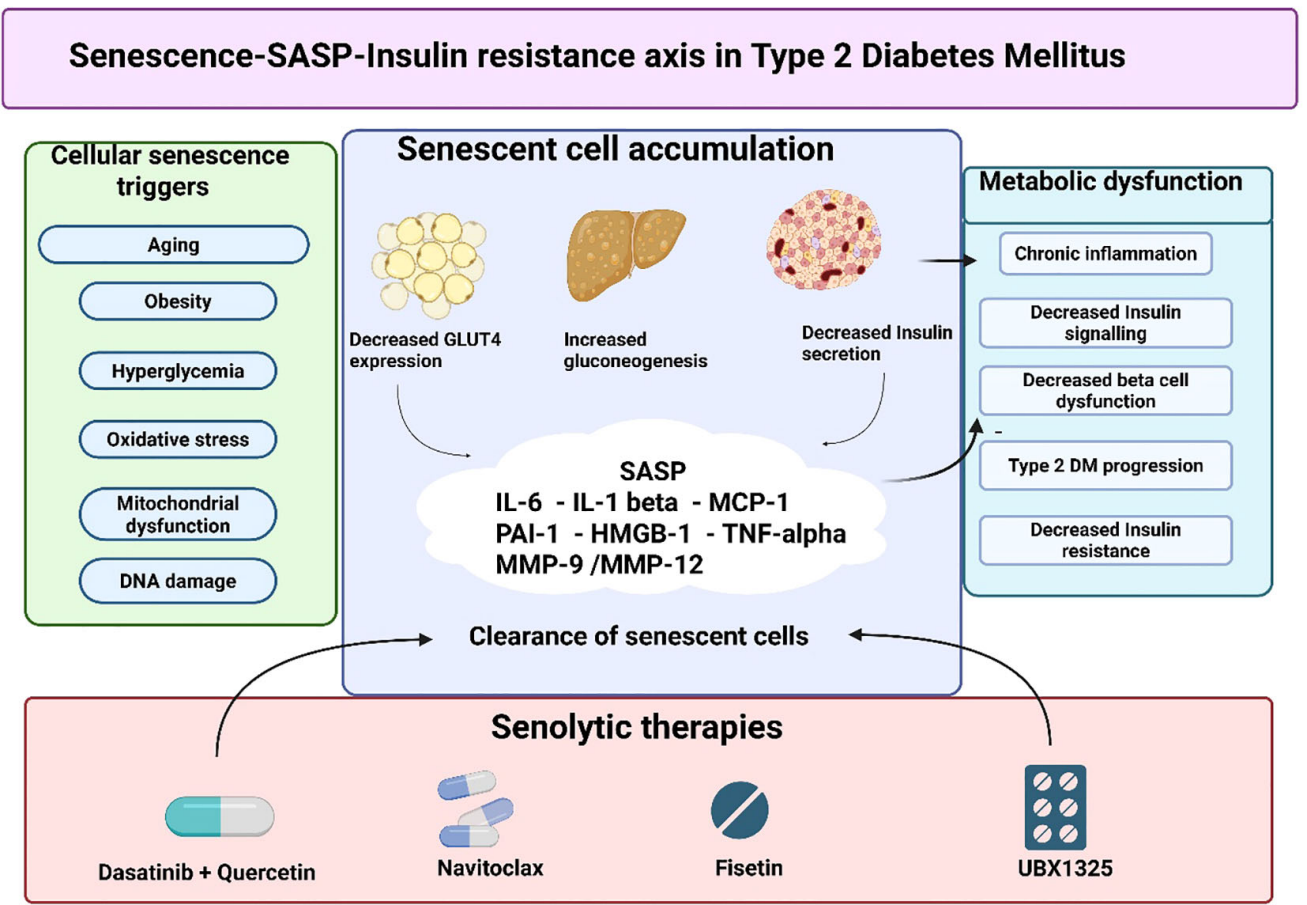
Senescence–SASP–insulin resistance axis in type 2 diabetes mellitus. Chronic metabolic stressors such as aging, obesity, hyperglycemia, oxidative stress, and mitochondrial dysfunction promote the accumulation of senescent cells in key metabolic tissues, including adipose tissue, liver, and pancreatic β-cells. These senescent cells undergo stable cell-cycle arrest while remaining metabolically active and secrete a senescence-associated secretory phenotype (SASP) composed of pro-inflammatory cytokines (IL-6, IL-1β, TNF-α), chemokines (MCP-1), proteases (MMP-9, MMP-12), and alarmins (HMGB1). Persistent SASP signaling drives chronic low-grade inflammation, disrupts insulin receptor substrate-1 (IRS-1) signaling, impairs adipocyte function, promotes hepatic gluconeogenesis, and accelerates β-cell dysfunction, thereby contributing to the initiation and progression of type 2 diabetes mellitus. Senolytic therapies selectively eliminate senescent cells by targeting senescent cell anti-apoptotic pathways (SCAPs), leading to attenuation of SASP burden, reduction of metabolic inflammation, and partial restoration of insulin sensitivity and β-cell function. This framework highlights cellular senescence as a mechanistically distinct and potentially disease-modifying therapeutic target in T2DM. The mechanistic pathways illustrated are supported by prior experimental and translational studies of SASP-mediated insulin resistance and β-cell dysfunction (5, 12–14). TAFs, Telomere-associated foci; SCAPs, senescent cell anti-apoptotic pathways, SASP - senescence-associated secretory phenotype.

As illustrated in **Figure 1**, metabolic stressors—including aging, obesity, chronic hyperglycemia, oxidative stress, and mitochondrial dysfunction—drive the accumulation of senescent cells within adipose tissue, liver, and pancreatic β-cells (15). These senescent cells persist due to activation of senescent cell anti-apoptotic pathways (SCAPs), allowing them to evade immune clearance and progressively accumulate in tissues critical for glucose regulation (16).

Importantly, even a relatively small burden of senescent cells can exert disproportionate metabolic effects through SASP-mediated signaling, establishing cellular senescence as a mechanistically distinct contributor to insulin resistance and β-cell dysfunction (12). This biological framework provides the rationale for senolytic therapies, which aim to selectively eliminate senescent cells rather than merely suppress downstream inflammation (17) (**Figure 1**).

### 3.3 Senescence-associated secretory phenotype as a driver of insulin resistance

The SASP is a complex mixture of pro-inflammatory cytokines (IL-6, IL-1β, TNF-α), chemokines (MCP-1), proteases (MMP-9, MMP-12), pro-fibrotic mediators (PAI-1), and alarmins such as HMGB1 (11). As outlined in **Figure 1**, sustained SASP signaling is the key mechanism contributing to the relationship between senescent cell accumulation and metabolic pathologic processes (6).

In adipose tissue, SASP factors recruit and activate macrophages, trigger local inflammation and dysregulated adipocyte differentiation leading to decrement of GLUT4 expression and insulin-stimulated glucose uptake (11). In the liver, SASP-mediated inflammation induces hepatic insulin resistance and enhances gluconeogenesis, which, in turn, mediates fasting hyperglycemia. Chronic SASP cytokine exposure in pancreatic islets enhances the onset of β-cell stress, functional exhaustion, and apoptotic loss (15). Together, these effects intersect with inhibition of insulin receptor substrate-1 (IRS-1) signaling and sustained low-grade inflammation (“inflammaging”), a feature of type 2 diabetes mellitus (T2DM). In this way SASP acts as a system-wide amplifier of metabolic dysfunction as opposed to being a local inflammatory phenomenon (13).

### 3.4 Triggers and heterogeneity of senescence in metabolic tissues

The induction of cellular senescence in metabolic tissues is multifactorial and context dependent (15). While chronological aging remains a dominant driver, metabolic stressors such as obesity, hyperinsulinemia, oxidative damage, mitochondrial dysfunction, and DNA damage can induce premature senescence independent of age (15). As summarized schematically in **Figure 1**, these triggers converge across adipose tissue, liver, and pancreatic β-cells, leading to tissue-specific but interconnected metabolic consequences (18, 19).

Senescent cells arise transiently and are efficiently cleared by immune surveillance, whereas others persist, particularly in the setting of metabolic disease where immune function is impaired. This persistence allows chronic SASP signaling to dominate tissue biology, tipping the balance from adaptive stress responses toward pathological inflammation and metabolic decline (20).

Meanwhile, senescence retains essential physiological roles in tumor suppression, tissue remodeling, and wound healing, underscoring the need for selective—not indiscriminate—therapeutic targeting. This duality reinforces the appeal of senolytic strategies designed to eliminate harmful senescent cell populations while minimizing disruption of beneficial senescence programs (21–23).

### 3.5 Role of senescent cells in insulin resistance and β-cell dysfunction

The accumulation of senescent cells within adipose tissue and pancreatic islets is increasingly recognized as a causal contributor to the two defining features of T2DM: insulin resistance and β-cell dysfunction. As shown in **Figure 1**, SASP-driven inflammation acts as a common upstream mechanism linking these processes across tissues (6).

In adipose tissue, senescent preadipocytes impair adipogenesis, reduce insulin responsiveness, and promote ectopic lipid deposition in liver and skeletal muscle, exacerbating systemic insulin resistance. Elevated circulating levels of SASP components such as IL-6, IL-1β, MCP-1, and PAI-1 correlate with increased risk of T2DM and its vascular complications (24–27).

Within pancreatic islets, β-cell senescence is characterized by upregulation of cell-cycle inhibitors (p16^INK4a, p21^Cip1), reduced insulin secretory capacity, and impaired regenerative potential. Experimental models demonstrate that clearance of senescent β-cells improves insulin secretion and glucose tolerance, providing direct evidence that senescence contributes to β-cell failure rather than representing a passive marker of disease progression (14, 28–30).

### 3.6 Evidence of cellular senescence in type 2 diabetes

A growing body of evidence shows that, as well as being found in metabolically active tissues, senescent cells are responsible for the pathogenesis and progression of type 2 diabetes mellitus (T2DM) (31). The accumulation of senescent cells in β-cells (32), adipose tissue (33), liver (34), and skeletal muscle (35) hinders metabolic stability, causes tissue inflammation due to its senescence, and intensifies insulin resistance. Further human studies have verified that senescent cell markers such as p16^INK4a, p21^Cip1, and SA-β-gal are consistently elevated in the tissues of people with diabetes and obesity, supporting their importance as disease markers (19, 36) (**Table 1**).

**Table 1:** Comparative tissue-specific patterns of cellular senescence and senolytic responsiveness in metabolic disease.

Tissue/organ	Key findings	Senescence markers	Senolytic interventions	Therapeutic outcomes	References
Pancreatic β-cells	Insulin resistance induces β-cell senescence, contributing to T2DM pathogenesis. Clearance of senescent cells improves glucose metabolism and insulin secretion.	p16INK4a, SASP, SA-β-gal	Navitoclax (ABT263); Dasatinib + Quercetin (D+Q)	Improved hyperglycemia, restored β-cell gene expression, reduced blood glucose levels	(32)
Adipose Tissue	Hyperinsulinemia triggers senescence in adipocytes and preadipocytes, leading to inflammation via SASP. Senolytics reduce senescent cell burden.	SA-β-gal, p16INK4a, p21Cip1, γH2AX, cyclin D1, loss of HMGB1	Dasatinib + Quercetin (D+Q)	Reduced p16INK4a and p21Cip1 cells, decreased SASP (IL-1α, IL-6, MMP-9, MMP-12), correlated with insulin resistance markers	(33)
Liver	Fat accumulation promotes hepatocyte senescence via telomere shortening and DNA damage. Senolytics reduce senescence and SASP.	p21Cip1, p53, SA-β-gal, telomere shortening	Dasatinib + Quercetin (D+Q)	Reduced hyperinsulinemia-induced senescence, decreased SASP, potential for NAFLD/NASH treatment	(34)
Skeletal Muscle	Senescence driven by oxidative stress, inflammation, and mechanical load. Senolytics improve muscle function.	p16INK4a, p21Cip1, SA-β-gal, SASP	Navitoclax (ABT263); Dasatinib + Quercetin (D+Q); Nicotinamide riboside	Reduced SASP, improved treadmill performance, enhanced myogenesis	(35)
Multiple Tissues	Senescent cells contribute to age-related pathologies in T2DM across tissues (e.g., adipose, liver, pancreas, muscle). Clearance extends median lifespan in mice.	SA-β-gal, p16INK4a, TAFs, DDR	INK-ATTAC; Dasatinib + Quercetin (D+Q)	Alleviated renal/hepatic dysfunction, pancreatic degeneration, neurodegeneration, improved physical strength	(19, 36)

This table contrasts how senescence markers, dominant SASP features, and senolytic responses differ across pancreatic, adipose, hepatic, and skeletal muscle tissues, illustrating tissue-specific vulnerability and therapeutic implications.

## 4 Senolytics: an emerging therapeutic class

### 4.1 Senolytics and their mechanisms of action

**Figure 2:**
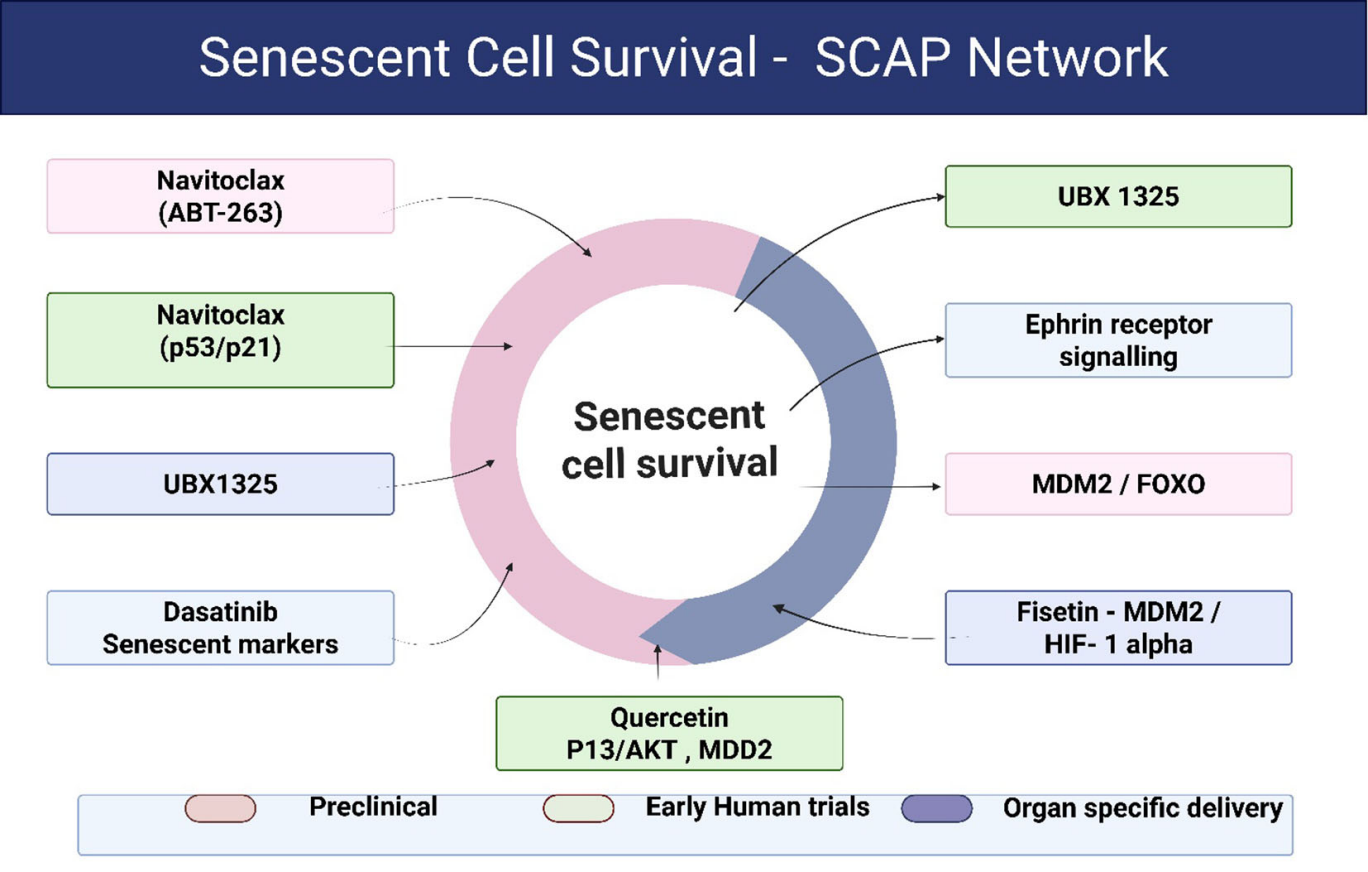
Senolytic drug classes and targeted senescent cell anti-apoptotic pathways (SCAPs). Senescent cells evade apoptosis through activation of senescent cell anti-apoptotic pathways (SCAPs), including BCL-2/BCL-XL, PI3K/AKT, p53/p21, and ephrin-mediated survival signaling. Senolytic agents selectively disrupt these pathways, triggering apoptosis in senescent cells while largely sparing healthy, proliferating cells. Synthetic agents such as navitoclax and UBX1325 primarily target BCL-xL-dependent survival, whereas dasatinib interferes with tyrosine kinase–associated SCAP signaling. Natural senolytics such as quercetin and fisetin exert pleiotropic effects across PI3K/AKT and oxidative stress–responsive pathways. The diversity of SCAP targeting highlights both the therapeutic promise and toxicity considerations of senolytic strategies in metabolic disease. SCAP pathway targeting mechanisms depicted are derived from experimental senolytic studies and pathway analyses described in prior mechanistic investigations (36, 41–44).

This shift from molecular description to biological integration allows cellular senescence to be interpreted not as a secondary epiphenomenon, but as a primary disease−organizing process in type 2 diabetes mellitus. Senolytics work by selectively clearing senescent cells through the disruption of senescent cell anti-apoptotic pathways (SCAPs), which are molecular survival systems that allow these cells to evade programmed cell death and continue accumulating in tissues (37, 38). Using RNA interference and large-scale transcriptomic screening, researchers have identified several major SCAP components, including BCL-2/BCL-XL, PI3K/AKT, and p53/p21/serpin signaling. These pathways play a central role in maintaining the survival of senescent cells and have therefore become attractive therapeutic targets (36, 39). Based on these discoveries, a new generation of senolytic drugs such as Dasatinib, Quercetin, Navitoclax, and Fisetin has been developed to trigger apoptosis specifically in senescent cells while sparing normal, healthy ones (40) (**Figure 2**, **Table 2**). The following section provides an overview of these agents and summarizes the most recent preclinical and clinical studies exploring their role in type 2 diabetes mellitus and its related complications (**Table 2**).

**Table 2:** Comparative targeting of senescent cell anti-apoptotic pathways by senolytic agents.

Senolytic agent	Targeted SCAP pathway	Mechanism of action	Key findings	References
Navitoclax (ABT263)	BCL-2/BCL-XL	Inhibits anti-apoptotic proteins, promoting senescent cell apoptosis	Improved glucose metabolism and β-cell function; restored gene expression in insulin-resistant models	(41)
Dasatinib	p53/p21, Ephrin receptors	Tyrosine kinase inhibition; disrupts SCAP pathways	Reduced β-cell and adipose tissue senescence; decreased SASP and inflammatory cytokines	(42)
Quercetin	PI3K, AKT, MDM2, P53, HIF-1α	Natural flavonoid targeting multiple SCAP pathways	Synergistic effect with Dasatinib; reduced p16INK4a and p21CIP1 expression; lowered SASP factors	(43)
Dasatinib + Quercetin (D+Q)	Combined SCAP inhibition	Targets a broader range of senescent cells by complementary pathway inhibition	Significant reduction in senescent cells in adipose tissue and skin; lower IL-1α, IL-6, MMP9, MMP12 levels	(44)
INK-ATTAC Mouse Model	p16Ink4a-positive cells	Genetic removal of senescent cells using inducible caspase-8 system	Improved insulin sensitivity and pancreatic function; delayed aging-related decline	(45)
Fisetin	PI3K/AKT pathway	Inhibiting antiapoptotic pathway and suppresses SASP	Reduced the risk of vascular and ischemic heart disease with a positive reduction in senescence	(46)

This table highlights mechanistic diversity, pathway selectivity, and translational constraints of major senolytic agents, rather than reiterating descriptive pharmacologic properties discussed in the text.

### 4.2 Potential of senolytics in combination therapy

Combination approaches pairing senolytic agents with established antidiabetic drugs have emerged as a novel therapeutic avenue, aiming to address both metabolic dysfunction and cellular senescence. One of the most promising strategies under exploration involves metformin combined with senolytics, particularly fisetin or dasatinib plus quercetin (D+Q). Metformin, an FDA-approved biguanide, enhances insulin sensitivity, suppresses hepatic gluconeogenesis, and downregulates IGF-1 and mTOR signaling, while inhibiting mitochondrial complex I to reduce endogenous reactive oxygen species (ROS) generation. Preclinical evidence indicates that combining metformin with senolytic agents augments suppression of the senescence-associated secretory phenotype (SASP) and improves mitochondrial function in diabetic models (47). On the other hand, no human clinical trials have yet evaluated this combination, and potential pharmacodynamic overlaps raise uncertainty regarding safety. The U.S. Food and Drug Administration (FDA) currently classifies senolytic-metformin combinations as investigational under metabolic indications, pending formal Phase I-II trials. Another proposed regimen combines GLP-1 receptor agonists (e.g., liraglutide, semaglutide) with D+Q, leveraging enhanced glucose-dependent insulin secretion, reduced glucagon output, and weight loss alongside senolytic clearance of senescent adipocytes. Preclinical models have demonstrated improvements in B-cell function, decreased inflammatory cytokines, and enhanced metabolic resilience when these therapies are co-administered (5, 48). However, overlapping gastrointestinal adverse effects may reduce tolerability, and thiscombination remains untested in human subjects. Regulatory authorities, including the FDA and European Medicines Agency (EMA), currently consider such studies exploratory and have not granted approval for clinical evaluation. A third experimental combination involves Navitoclax, a BCL-2/BCL-xL inhibitor, used alongside SGLT2 inhibitors such as empagliflozin or dapagliflozin (49).

In animal models, this approach has been shown to induce apoptosis of senescent renal and adipose cells, decrease inflammation, and improve insulin sensitivity while simultaneously lowering blood glucose through renal glucose reabsorption blockade (50). However, translational progress is limited by Navitoclax-associated thrombocytopenia and neutropenia, adverse effects identified in early oncology trials. Both the FDA and EMA currently restrict Navitoclax to oncology investigational new drug (IND) programs due to these hematologic toxicities, underscoring the challenge of adapting it for metabolic applications. Another combination of interest is DPP-4 inhibitors (e.g., sitagliptin, linagliptin) with natural senolytics such as fisetin, which elevate incretin levels, enhance insulin secretion, and suppress glucagon, thereby improving overall glycemic control (51). Preclinical evidence suggests that combining DPP-4 inhibitors with fisetin amplifies anti-inflammatory and antioxidative effects in diabetic models. However, fisetin’s poor oral bioavailability and limited pharmacokinetic data hinder clinical translation, and the compound remains classified as a nutraceutical rather than a therapeutic agent by both FDA and EMA.

In summary, while these combination therapies represent a forward-looking strategy for integrating senotherapeutic and metabolic interventions, translation into clinical practice remains constrained by several key factors including but not limited to; the absence of long-term placebo-controlled studies, small and heterogeneous preclinical cohorts, and lack of validated glycemic endpoints.

## 5 Senolytic therapy in diabetes: preclinical and clinical insights

Human studies evaluating senolytic therapy in T2DM should be interpreted strictly as early-phase translational investigations. None were designed or powered to assess glycemic efficacy, disease modification, or long-term metabolic outcomes.

### 5.1 Animal models of T2DM treated with senolytics

Recent publications conducted on the therapeutic effects of senolytic agents on type 2 diabetes animal models showed a positive correlation in alleviating the symptoms and complications. In 2019, using a p16INK4a promoter potentially led to the improving of metabolic and adipose dysfunction (52). In 2022, another cocktail of drugs were used called mainly quercetin and dasatinib reviewed promising results in glucose and insulin resistance in obese mice which were transplanted with adipose tissue (53) while using a modified version of tamoxifen which targets mitochondria reduced appetite, adipogensis and senescent cells (54). Adding onto that, dasatinib when used has shown to reduce cardiac steatosis and fibrosis in diabetic mice models (55) and further studies conducted revealed improved wound healing with senolytic therapy on wound sites by inhibiting the senescent cell (56). *in vivo* and *in vitro* studies using fisetin had led to a positive effect in reducing aortic senescence and SASP factors (57).

#### 5.1.1 Limitations of preclinical senolytic models in metabolic disease

Although preclinical studies consistently demonstrate improvements in insulin sensitivity, inflammatory burden, and β-cell preservation following senescent cell clearance, several important limitations warrant consideration.

First, metabolic phenotypes vary substantially across mouse models, including diet-induced obesity, leptin-deficient (ob/ob), and streptozotocin-induced diabetic models. These models differ in inflammatory tone, β-cell reserve, and immune response, limiting cross-model comparability.

Second, senolytic dosing paradigms in murine studies frequently employ intermittent high-dose regimens that may not translate directly to human pharmacokinetic constraints. The temporal dynamics of senescent cell clearance and repopulation remain incompletely characterized in metabolic tissues.

Third, endpoints are heterogeneous, ranging from glucose tolerance tests to surrogate inflammatory markers, with inconsistent measurement of durable HbA1c equivalents or long-term β-cell mass preservation.

Fourth, certain senolytic agents—particularly navitoclax—demonstrate dose-dependent thrombocytopenia due to BCL-xL inhibition in platelets, highlighting potential off-target toxicity.

Collectively, these limitations underscore that while mechanistic plausibility is strong, preclinical findings should be interpreted cautiously when extrapolating toward human metabolic disease.

### 5.2 Clinical trials and human studies

#### 5.2.1 Systemic metabolic and biomarker studies- not powered for glycemic endpoints

There have been very few clinical trials conducted on humans that have been conducted that have concluded nonetheless, similar outcomes recorded in the animal models themselves. A very vital clinical trial conducted on humans was the use of a combination therapy of Dasatinib and Quercetin on obese patients with diabetic kidney disease which is a very common complication of long standing diabetes mellitus type 2, these participants received an oral therapy of the drugs for a course of 3 days and biopsies and blood samples were obtained 11 days after that to evaluate the effects (58). The results showed significant decrease in the burden exerted by the senescent cells on the subcutaneous adipose tissue, along with that it also showed reduced levels of senescence-associated secretory phenotype (SASP) factors circulating in the bloodstream which led us to infer that there is systemic anti-inflammatory benefits of using this drug and lastly, there were no side effects with the use of these drugs as it was a short course of treatment. Although the results seem very positive there are however a lot of limitations for the use of this study, the sampling size in which this therapy was used was only 9 people and there was no placebo kept for this specific research. Another very vital measurement left out was to check if the glycemia was controlled by the drug or not by checking the hbA1c or checking for insulin sensitivity. Last but not the least, this research mainly focuses on the complication and not on the effects it will have on diabetes type 2 mellitus as a whole.

Follow-up duration in available studies has ranged from 7 to 28 days, precluding assessment of sustained metabolic effects. Safety signals in early trials have generally been mild and transient; however, these studies were not powered to detect rare adverse events. Heterogeneity across trials reflects differences in patient populations (e.g., diabetic kidney disease vs. advanced age), dosing schedules, tissue endpoints, and absence of standardized metabolic outcome measures. These factors collectively limit interpretability and emphasize the need for harmonized trial design.

#### 5.2.2 Organ-specific senolytic investigations

Another research that is quickly gaining popularity is the use of foselutoclax (a novel UBX1325) as a single dose intravitreal injection for patients with Type 2 Diabetes Mellitus-associated macular edema, this trial revealed that not only did the patients tolerate the drug well with no other complications it also showed an improvement in the patient’s visual acuity (59). The limitations of using this study does resemble the same challenges we faced above, with a small sampling size it’s not possible to generalize the whole population based on them. **Table 3**. Adding onto that, the use of senolytics although may show benefit for a short term, but the use of long term therapy and its efficacy still remains in the grey as more research is yet to be done.

**Table 3:** Summary of clinical and translational studies evaluating senolytic therapies in metabolic disease.

Evidence level	Study type	Model/population	Senolytic intervention	Primary focus	Key findings	Translational status	Key limitations
Mechanistic	*In vitro*	Human and murine metabolic cells	Dasatinib, Quercetin, Navitoclax	SASP signaling and cell survival pathways	Reduction in SASP factors; restoration of insulin signaling pathways	Preclinical	Cell-based systems lack tissue and systemic context
Preclinical	Animal models	Obese and diabetic mouse models	Dasatinib + Quercetin; Navitoclax; Fisetin	Insulin sensitivity and β-cell function	Improved glucose tolerance; reduced inflammatory burden; partial β-cell preservation	Preclinical	Species-specific responses; dosing not directly translatable
Translational	Pilot clinical studies (Phase I)	Patients with T2DM and diabetic kidney disease	Dasatinib + Quercetin (short intermittent course)	Senescence biomarkers and SASP reduction	Decreased p16INK4a-positive cells and circulating inflammatory mediators	Early human validation	Small sample size; short duration; no glycemic endpoints
Organ-specific	Phase II randomized trials	Patients with diabetic macular edema	UBX1325 (BCL-xL inhibitor)	Organ-specific senescence outcomes	Improved visual acuity with acceptable short-term safety	Exploratory clinical	Non-glycemic endpoint; limited follow-up
Ongoing	Registered clinical trials	Older adults with metabolic disease	Fisetin; Navitoclax (investigational)	Safety and biomarker modulation	Trials ongoing; efficacy outcomes pending	Investigational	No published metabolic efficacy data yet

This study evaluated ocular structural and visual endpoints and did not assess systemic glycemic control or metabolic parameters.

Importantly, no senolytic therapy has yet demonstrated clinical efficacy in improving HbA1c, insulin sensitivity, or long-term metabolic outcomes in individuals with T2DM. Existing human studies should be interpreted as evidence of biological target engagement rather than proof of therapeutic benefit. At present, senolytic therapy remains an experimental geroscience-based strategy requiring rigorous validation in adequately powered metabolic trials.

### 5.3 Interpretive framework and evidence boundaries

Taken together, current human studies of senolytic therapy should be viewed as biological validation studies rather than efficacy trials. While reductions in senescence markers and inflammatory mediators are consistently observed, these surrogate outcomes do not yet translate into demonstrable improvements in glycemic control, insulin sensitivity, or β-cell preservation. The distinction between mechanistic promise and clinical effectiveness is critical, particularly in a heterogeneous disease such as T2DM.

Accordingly, senolytic therapy should presently be regarded as an experimental geroscience-based intervention, with potential relevance to metabolic disease that requires rigorous confirmation in adequately powered, randomized clinical trials using standardized metabolic endpoints. Until such data are available, claims regarding disease modification or glycemic benefit must remain provisional.

## 6 Integrative discussion and translational implications

This review synthesizes current evidence positioning cellular senescence as a biologically relevant contributor to metabolic dysfunction in T2DM. Rather than redefining T2DM exclusively as an aging disorder, we propose that senescence biology represents one upstream mechanistic layer interacting with established metabolic drivers such as obesity, insulin resistance, and glucotoxicity.

### 6.1 Integrative overview of mechanistic pathways

When viewed through the perspective of biological aging, type 2 diabetes mellitus (T2DM) is presented not only as a glucose metabolic disorder but also as a systemic condition, with a number of factors contributing to it including chronic cellular stress and maladaptive responses to aging. Cellular senescence presents an unifying mechanism, connecting metabolic overload, chronic inflammation, and tissue dysfunction in adipose tissue, liver, skeletal muscle, and pancreatic islets.

Rather than separate molecular anomalies, senescence-related pathways come together at the level of three interrelated mechanisms—sustained SASP-dependent inflammation, disruption of insulin receptor signaling, and impaired cellular regeneration. Several pro-inflammatory cytokines including IL-6, IL-1β, and TNF-α suppress insulin receptor substrate-1 (IRS-1) signaling, whereas chemokines and matrix-remodeling enzymes modify tissue architecture and enhance immune cell infiltration. In pancreatic β-cells, senescence limits proliferative capacity and accelerates functional exhaustion, amplifying glycemic dysregulation (**Table 4**).

**Table 4:** Senescence-associated molecular pathways linking inflammation and metabolic dysfunction.

Pathway	Representative molecules	Metabolic consequences	Theraputic implication
Pro-inflammatory cytokine axis	IL-6, IL-1β, TNF-α, MCP-1	Inhibits insulin receptor substrate (IRS-1) signaling; promotes hepatic gluconeogenesis	Targeted by senolytics and senomorphics to attenuate chronic inflammation
Fibrotic/remodeling mediators	MMP-9, PAI-1, TGF-β	Alters extracellular matrix and adipose remodeling, impairing insulin sensitivity	Reduced after D + Q or Fisetin therapy
Alarmin-oxidative stress loop	HMGB1, ROS, RAGE	Propagates mitochondrial stress, β-cell apoptosis	Potential target for antioxidant-based senomorphics (e.g., metformin, resveratrol)

### 6.2 Clinical evidence in context: from mechanistic promise to translational caution

When interpreted within this aging-centered framework, the current human evidence for senolytic therapy assumes appropriate—but limited—significance. Existing clinical studies demonstrate biological target engagement, including reductions in senescence markers and circulating SASP components, thereby validating the mechanistic plausibility of senescent cell clearance in humans.

However, none of the available trials were designed to assess disease modification in T2DM. Sample sizes remain small, treatment durations short, and endpoints largely restricted to surrogate biomarkers or organ-specific outcomes. Importantly, no study has yet demonstrated durable improvements in HbA1c, insulin sensitivity, or β-cell preservation.

This distinction between mechanistic validation and clinical efficacy is critical. While senolytic therapy aligns conceptually with the aging-driven model of T2DM, its therapeutic relevance remains hypothetical until confirmed by adequately powered, randomized trials with standardized metabolic endpoints. The absence of proven glycemic efficacy should not be interpreted as failure of the senescence framework, but rather as a reflection of the early translational stage of this field (**Table 5**).

**Table 5:** Registered clinical trials investigating senolytic therapies in metabolic and age-related conditions.

Trial ID	Phase/status	Intervention	Population	Primary endpoint
NCT02848131	Phase I/II – Completed	Dasatinib + Quercetin	Idiopathic pulmonary fibrosis with metabolic comorbidities	Safety/senescence biomarkers
NCT04685590	Phase II – Recruiting	UBX1325 (BCL-xL inhibitor)	Diabetic macular edema	Visual acuity/ocular senescence markers
NCT05249845	Phase I – Recruiting	Fisetin (oral, 100 mg/day)	Older adults with T2DM	Changes in inflammatory cytokines and HbA1c
NCT05023682	Phase I – Active	Navitoclax	Advanced metabolic syndrome	Dose-limiting toxicity and pharmacokinetics

Collectively, these trials emphasize early safety and biomarker modulation, but none yet provide large-scale glycemic efficacy data or standardized metabolic endpoints.

### 6.3 Pharmacodynamic and off-target considerations in an aging biology context

Targeting senescent cells introduces unique pharmacodynamic challenges that differ fundamentally from conventional antidiabetic therapies. Senolytics exert short-lived systemic exposure but induce prolonged biological effects through irreversible elimination of senescent cells. While this intermittent dosing paradigm is theoretically attractive, it also magnifies the importance of selectivity.

Early-generation senolytics such as navitoclax illustrate the tension between efficacy and toxicity, as BCL-xL inhibition affects both senescent cells and platelets. Similarly, dasatinib and quercetin display pleiotropic actions that may influence immune surveillance, mitochondrial function, and tissue repair. These considerations underscore that senescence is not uniformly pathological; it also plays essential roles in wound healing, tumor suppression, and immune regulation.

Consequently, therapeutic strategies must prioritize selective senescent cell targeting, tissue specificity, and intermittent exposure to minimize unintended disruption of beneficial senescence programs—particularly in older adults with multiple comorbidities.

Pharmacokinetic considerations are particularly relevant in T2DM populations characterized by polypharmacy, chronic kidney disease, hepatic steatosis, and cardiovascular comorbidities. Dasatinib is metabolized via CYP3A4, raising potential drug–drug interaction concerns with commonly prescribed statins, calcium channel blockers, and certain antihyperglycemic agents. Navitoclax-associated thrombocytopenia may be amplified in patients receiving antiplatelet or anticoagulant therapy, which are frequently used in individuals with diabetes and established atherosclerotic disease. Furthermore, altered renal clearance and hepatic metabolic capacity in diabetes may affect senolytic drug exposure, underscoring the need for population-specific dosing studies and formal pharmacokinetic evaluation in metabolically compromised patients.

### 6.4 Senolytics versus senomorphics: distinct roles in diabetes management

Within the aging-centered paradigm of T2DM, senolytics and senomorphics should be viewed as complementary rather than competing strategies. Senolytics aim to reduce senescent cell burden through targeted apoptosis, potentially resetting inflammatory and metabolic set points. In contrast, senomorphics suppress SASP production and modulate senescent cell behavior without inducing cell death.

Widely used antidiabetic agents such as metformin, GLP-1 receptor agonists, and SGLT2 inhibitors exhibit senomorphic properties, including attenuation of oxidative stress, enhancement of autophagy, and modulation of inflammatory signaling. These agents may therefore serve as long-term modulators of inflammaging, while senolytics—if proven safe and effective—could be deployed intermittently in carefully selected patients to reduce pathological senescent cell accumulation.

The **Table 6** integrates molecular senescence pathways with corresponding metabolic consequences and therapeutic strategies, highlighting the mechanistic rationale, pharmacologic targeting approaches, and current translational maturity of senolytic and senomorphic interventions in type 2 diabetes mellitus.

**Table 6:** Integrated mechanistic–strategic framework linking senescence pathways to therapeutic targeting in type 2 diabetes mellitus.

Senescence-associated pathway	Representative molecular drivers	Metabolic consequences in T2DM	Therapeutic strategy	Representative agents	Translational status
Pro-inflammatory SASP axis	IL-6, IL-1β, TNF-α, MCP-1	Inhibition of IRS-1 signaling; increased hepatic gluconeogenesis; systemic insulin resistance	Senolytics (cell clearance) and Senomorphics (SASP suppression)	Dasatinib + Quercetin; Fisetin; Metformin	Early human biomarker studies; no glycemic efficacy data
Fibrotic and matrix remodeling pathway	MMP-9, MMP-12, PAI-1, TGF-β	Extracellular matrix remodeling; adipose dysfunction; β-cell microenvironment disruption	Predominantly Senolytics; partial modulation via senomorphics	D+Q; Navitoclax; GLP-1 receptor agonists (indirect anti-inflammatory effects)	Preclinical dominant; limited organ-specific human trials
Alarmin–oxidative stress loop	HMGB1, ROS, RAGE activation	Mitochondrial dysfunction; β-cell apoptosis; impaired regenerative capacity	Primarily Senomorphics; adjunct antioxidant approaches	Metformin; NAD^+^ boosters; Resveratrol	Clinically established metabolic agents with geroprotective signals
SCAP survival signaling	BCL-2/BCL-xL, PI3K/AKT, p53/p21	Persistence of senescent cell burden; chronic SASP amplification	Direct Senolytic targeting	Navitoclax; UBX1325; Dasatinib	Experimental; safety and selectivity concerns
Regenerative impairment pathway	p16INK4a, p21Cip1 upregulation	Reduced β-cell proliferative reserve; impaired tissue renewal	Precision-based intermittent senolysis	Investigational senolytics	Hypothesis-generating; requires randomized metabolic trials

### 6.5 Translational outlook

Moving senolytic treatment from experimental geroscience to clinical diabetology will require a precision-medicine framework combining biological aging metrics with metabolic phenotyping. At the center of the endeavor is the development of validated senescence biomarkers that can quantify senescent cell burden, monitor therapeutic response, and differentiate pathological from adaptive senescence.

Integrated multi-omics techniques—transcriptomic, proteomic, and metabolomic profiling—fused with artificial intelligence (AI)-powered analytics present a valuable suite of tools for patient stratification, dose optimization, and toxicity prediction. Such approaches may also allow the identification of those for whom senescence-driven pathology predominates, maximizing benefit and minimizing risk.

In the end, large-scale randomized clinical trials incorporating aging-informed endpoints will ascertain if senolytic therapy can bring about meaningful alteration in the natural history of T2DM. Until such evidence emerges, senolytics should be considered investigational agents that shed light on a novel disease framework rather than established therapeutic.

## 7 Challenges and considerations

Senolytic therapy when used as a modality of treatment in treating type 2 diabetes mellitus does also pose serious challenges and considerations that we have to take into account when using them and as mentioned above there isn’t much research done or trials conducted to further evaluate the potential challenges that have been faced and how to overcome these obstacles, the **Table 7** further explains it.

**Table 7:** Translational, regulatory, and risk–benefit considerations for senolytic therapies in metabolic aging.

Challenge / Consideration	Potential solution / Innovative approach	Evidence / References	Regulatory and policy perspective (FDA / EMA)	Risk–benefit framework
1. Limited human clinical evidence	Conduct multicenter Phase II–III randomized controlled trials (e.g., Dasatinib + Quercetin) to establish long-term efficacy, optimal dosing, and safety.	(59, 60)	FDA: Encourages IND submissions under metabolic / geroscience indications. EMA: Supports adaptive-pathway pilot trials once validated biomarkers are established.	Benefit: Proof-of-concept efficacy. Risk: Sparse longitudinal data and uncertain chronic safety.
2. Off-target toxicity of early-generation senolytics	Develop mitochondria-targeted, self-assembling senolytic systems (integrin αvβ3, ROS-triggered activation) for selective elimination.	(61, 62)	Regulators require tissue-specific selectivity and standardized toxicity assays before pivotal studies.	Benefit: Enhanced specificity. Risk: Potential mitochondrial stress in healthy cells.
3. Non-specific drug release injuring normal cells	Engineer smart nanodevices (e.g., Navitoclax nanocarriers) activated by senescence-associated MMP-3 or ionizing-radiation cues.	(63)	FDA / EMA: Stress validation of trigger mechanisms and low systemic exposure.	Benefit: Controlled release limits collateral damage. Risk: Nanomaterial immunogenicity or instability.
4. Difficulty distinguishing senescent vs. quiescent cells	Apply Conditionally Active Biologics (CAB) targeting inflammatory microenvironments to spare non-senescent cells.	(64)	Agencies recommend harmonized senescence-specific assays and avoidance of quiescent-cell depletion.	Benefit: Improved target precision. Risk: Context-dependent activation may limit efficacy.
5. Suboptimal drug delivery and stability	Use “double-lock” nanoplatforms (β-galactosidase + acidic pH triggers) and micelle nanocarriers (e.g., GL392) for controlled release and enhanced stability.	(65–67)	Regulatory focus on reproducibility, nanocarrier stability, biodistribution, and manufacturing compliance (CMC).	Benefit: Improved pharmacokinetics and targeting. Risk: Complex production and cost.
6. Off-target effects and systemic toxicity	Employ galactose-conjugated senolytic prodrugs cleaved only by senescence-associated biomarkers.	(66)	Regulators require companion-diagnostic validation and risk-management monitoring.	Benefit: Reduced systemic toxicity. Risk: Inter-patient biomarker variability.
7. Limited therapeutic scope beyond senolysis	Utilize pleiotropic agents (e.g., Quercetin) for antioxidant, neuroprotective, and anti-inflammatory effects.	(60)	Broader indication acceptable if mechanism and safety are well defined.	Benefit: Broader metabolic protection. Risk: Mechanistic ambiguity in efficacy.
8. Need for improved stability and efficacy	Combine lipofuscin-binding scaffolds with micelle nanocarriers to optimize bioavailability and potency.	(67)	Regulatory bodies expect proof of formulation stability and cross-species safety.	Benefit: Sustained efficacy with lower doses. Risk: Unknown long-term tissue accumulation.
9. Regulatory uncertainty and lack of harmonized biomarker frameworks	Develop multi-omics and AI-based models to define senescence signatures and enable precision stratification.	—	FDA / EMA: Jointly promote AI-assisted biomarker qualification, adaptive trials, and post-market pharmacovigilance.	Benefit: Accelerated approval and better patient selection. Risk: Data-privacy and model-transparency issues.
10. Comprehensive risk–benefit assessment for translation	Integrate precision-medicine analytics (genomics, proteomics, metabolomics, AI) to predict responders and adverse events.	—	Supported under the emerging Geroscience Regulatory Framework and adaptive licensing schemes.	Overall Benefit: Reduced senescence burden, better metabolic control, health-span extension. Overall Risk: Uncertain chronic toxicity, cost, and ethical implications of lifespan modulation.

### 7.1 Translational and regulatory changes

Translation of senolytic therapies into clinical diabetology faces several regulatory and practical challenges. Current human evidence remains limited to small, early-phase studies primarily evaluating safety and biomarker modulation. Larger multicenter randomized trials are required to establish long-term efficacy, optimal dosing strategies, and metabolic endpoints relevant to T2DM.

Off-target toxicity remains a concern, particularly with first-generation agents such as navitoclax, where thrombocytopenia has limited broader clinical development. Regulatory authorities appropriately require demonstration of tissue selectivity, reproducible biomarker validation, and acceptable safety margins before advancing metabolic indications.

Additional challenges include the absence of standardized senescence biomarkers, heterogeneity of T2DM phenotypes, and the need for precision-based patient stratification. While emerging approaches involving multi-omics profiling and AI-assisted analytics may support future trial design, these remain investigational and should be interpreted cautiously.

Accordingly, senolytic therapy currently occupies an exploratory position within geroscience-informed endocrinology rather than an established therapeutic pathway.

## 8 Safety and ethical concerns

Although there has been extensive light shed on senolytic therapy for its potential to treat Type 2 Diabetes Mellitus, there has however been a lot of concerns to address based on its safety and its toxicity side effects. A research concluded that when an older mouse model infected in Influenza A virus treated with any senolytic therapy like dasatinib and quercetin showed no improvement in survival or weight loss which led us to infer that senolytic treatments may not pose any effect if used on elderly with associated influenza A virus. Additionally, the research also concluded that when treatment like dasatinib and quercetin were administered it further suppressed the immune cell infiltration so it also led us to infer that it may exacerbate the immune response (68). Although the benefits do seem to outweigh the risk, since this discovery is relatively new and does not have much evidence to back the long term use of these therapies, the variability of senescent cell reaction to the drug differs drastically from species to species (69). Another potential concern to address is informed consent, as using these newly discovered drugs may have unknown side effects not yet studied and this in turn makes it harder to obtain informed consent in vulnerable populations like patients with diabetes (70). A very vital problem to tackle is the cost incurred on the patients end, as senolytics is a new branch in medicine the cost is usually higher when compared to other drugs. This in turn may lead to a disparity that the low-income groups cannot afford and raises ethical concerns based on fairness in treating the same condition (71). Another ethical concern does arise when we talk about unintended physiological consequences of using this therapy. Senescent cells play a crucial role in maintaining the immune system and in tissue repair, the removal of these may result in an increase in the chance of getting cancer or may cause immunosuppression. This could potentially pose ethical dilemmas about the harm versus the benefit of the use of senolytic therapy in treating Type 2 Diabetes Mellitus.

## 9 Future directions and research gaps

Senolytic therapy is an emerging field with promising but preliminary evidence to suggest the benefits, and significant knowledge gaps remain before clinical application. Preclinical and early clinical findings highlight the clear need for large-scale stage II, III randomized controlled trials to clinically establish efficacy in humans and tailor doses as well as test long-term safety. While short-term advantages have been repeatedly reported, the long-term safety of senolytics is still unknown, including drug–drug interaction sparing and their effects on individuals with multiple comorbidities.

One of the obstacles in this respect is that no universal biomarkers are available to precisely measure senescent cell burden, follow therapeutic progress and apply precision medicine. Since T2DM is a heterogeneous disease so one size does not fit all will be ideal. A multi-omics and artificial intelligence (AI)-powered approach, instead, is more likely to be recognized as crucial for uncovering senescence-related molecular signatures and stratifying patients into biologically relevant subclasses. Integrative analysis of transcriptome, proteome, metabolome and epigenome data retrieved by machine learning models can further identify the hidden senescence-related patterns associated with metabolic impairment, inflammatory response and drug response. They have the potential not only to provide fine-tuning of patient selection for senolytic therapy but also to discover new therapeutic and predictive targets.

Therefore, the future of senolytic therapy in diabetes rests on a multidisciplinary precision-medicine approach although currently senolytic therapy is currently being viewed as a biologically informative experimental strategy that advances disease modeling and patient stratification paradigms, rather than a clinically deployable treatment modality.

## 10 Biological and clinical uncertainties

While accumulating evidence supports the mechanistic plausibility of targeting senescent cells in metabolic disease, several biological uncertainties warrant careful consideration.

First, senescence is context-dependent and may exert protective roles in tumor suppression, wound healing, and tissue remodeling. Indiscriminate clearance of senescent cells could theoretically impair regenerative responses or immune surveillance.

Second, certain models suggest that transient senescence may facilitate adaptive metabolic remodeling. Complete ablation may therefore not uniformly confer benefit.

Third, immune modulation following senolytic exposure remains incompletely understood, particularly in older adults with multimorbidity. Potential risks include impaired pathogen defense or altered oncologic risk.

Fourth, several preclinical studies have demonstrated modest or variable metabolic improvements, indicating that senescence may represent one contributory mechanism rather than a universal driver of T2DM.

Accordingly, senolytic therapy must be evaluated within a rigorous risk–benefit framework, emphasizing selectivity, tissue targeting, and biomarker-guided stratification.

## 11 Conclusion

Cellular senescence represents a biologically relevant contributor to the pathogenesis of type 2 diabetes mellitus (T2DM), primarily through sustained senescence-associated secretory phenotype (SASP)-mediated inflammation and impaired tissue regenerative capacity. Chronic metabolic stressors—including aging, obesity, hyperglycemia, oxidative stress, and mitochondrial dysfunction—promote senescent cell accumulation in adipose tissue, liver, and pancreatic β-cells, where persistent SASP signaling disrupts insulin receptor pathways and accelerates β-cell functional decline. Preclinical models consistently demonstrate metabolic improvement following senescent cell clearance; however, current human evidence is limited to early-phase studies evaluating biomarker modulation and short-term safety rather than glycemic efficacy.

Despite compelling mechanistic plausibility, no senolytic intervention has yet demonstrated sustained improvement in HbA1c, insulin sensitivity, or long-term clinical outcomes in T2DM. Larger, adequately powered randomized trials incorporating standardized metabolic endpoints are required before therapeutic claims can be substantiated (72).
